# Biofilms as potential reservoirs of antimicrobial resistance in vulnerable settings

**DOI:** 10.3389/fpubh.2025.1568463

**Published:** 2025-03-21

**Authors:** Yanina Nahum, Johnathan Muhvich, José Rubén Morones-Ramirez, Nestor G. Casillas-Vega, Muhammad H. Zaman

**Affiliations:** ^1^Department of Biomedical Engineering, Boston University, Boston, MA, United States; ^2^Center on Forced Displacement, Boston University, Boston, MA, United States; ^3^Facultad de Ciencias Químicas, Universidad Autónoma de Nuevo León, Monterrey, Nuevo León, Mexico; ^4^Centro de Investigación en Biotecnología y Nanotecnología, Facultad de Ciencias Químicas, Universidad Autónoma de Nuevo León, Monterrey, Mexico; ^5^Departamento de Patología Clínica, Hospital Universitario Dr. José Eleuterio González, Universidad Autonoma de Nuevo León, Monterrey, Mexico

**Keywords:** antimicrobial resistance (AMR), wastewater surveillance, migrant shelters, drug-resistant organisms, gene transfer

## Abstract

Antimicrobial resistance is a major global health threat, characterized by the ability of microorganisms to withstand the effects of antimicrobial agents. Biofilms, as unique microbial communities, significantly contribute to this threat. They provide a protective environment for pathogens, facilitate horizontal gene transfer, and create an ideal setting for the persistence and evolution of resistant bacteria. This issue can be particularly important in low-income settings and vulnerable communities, such as formal and informal refugee and migrant camps. These settings usually have limited access to healthcare resources and appropriate treatments, contributing to the selective pressure that promotes the survival and proliferation of resistant bacteria. Thus, biofilms formed in wastewater in these areas can play a critical role in spreading antimicrobial resistance or acting as hidden reservoirs for future outbreaks. While emerging efforts focus on detecting antibiotic resistance genes and planktonic bacteria in wastewater, biofilms may be a source of under-appreciated antimicrobial resistance, creating a significant gap in our understanding of resistance dynamics in wastewater systems. Incorporating biofilm surveillance into wastewater monitoring strategies in vulnerable settings can help develop a more comprehensive understanding of resistance transmission and more effective intervention measures in these settings.

## 1 Introduction

Antimicrobial resistance (AMR) is a critical global health threat characterized by the ability of microorganisms to resist the effects of antimicrobial drugs. Especially during the last decade, there has been a dramatic increase in the number of multidrug-resistant bacteria (1), with estimates of up to 10 million deaths annually by 2050 (2). The cause of this emergence is related to several factors, including misuse and overuse of antimicrobials in humans and animals, mainly due to inadequate regulation of antibiotic use, leading to accumulation of antimicrobials in the environment and further enhancing resistance in microorganisms found in soil and water (3). The development of antibiotic-resistant bacteria primarily occurs after exposure to sub-inhibitory antibiotic doses because of the selective pressure these doses have on bacteria. The rapid spread of AMR is particularly pronounced in low-income settings–including urban slums, refugee camps, or forcibly displaced communities–where inadequate resources, high population density, exposure to infection and pathogens, limited or restricted access to healthcare, or incorrect treatment protocols contribute to accelerating the development and transmission of resistant pathogens (4).

### 1.1 Wastewater biofilms as reservoirs of AMR

A significant factor contributing to AMR is the presence of biofilms, which represent the preferred mode of life for bacteria and other microorganisms in wastewater environments. Biofilms are communities of microorganisms that generally grow attached to both biotic and abiotic surfaces and secrete a self-produced matrix of extracellular polymeric substances (EPS). The matrix provides a strong skeleton for biofilms to attach to pipes, tanks, and sediments, allowing them to persist over time. Additionally—and more critically—the EPS matrix acts as a barrier against the penetration of antibiotics or antimicrobials, providing a unique protective environment for microorganisms (5). The EPS matrix can also chemically adsorb and neutralize antimicrobial agents, reducing their efficacy and leading to sub-inhibitory concentrations of antibiotics within the biofilm. Other biofilm-mediated resistance mechanisms include quorum-sensing communication between cells to coordinate and synchronize gene exchange (6). These complex mechanisms within biofilms can significantly enhance resistance to antimicrobials—up to 1,000 times more than in planktonic cells—quickly, leading to the development of multidrug-resistant bacteria (7).

Wastewater, residential, industrial, or healthcare sewage, is often considered a reservoir of antibiotic-resistant genes (ARGs). These genes are fragments of DNA that bacterial communities can exchange to acquire resistance. One of the primary mechanisms enabling this exchange is horizontal gene transfer, either through direct contact (conjugation), uptake of free DNA from the environment (transformation), or transfer of DNA via bacteriophages (transduction) (4). Additionally, wastewater typically contains antibiotic residues from human and animal waste, and while wastewater treatment plants (WWTPs) are designed to reduce contaminants and improve water quality, conventional technologies are not fully equipped to remove low concentrations of pharmaceuticals—including antibiotics—leading to their release into surface waters. This can create a selective pressure that promotes the survival and proliferation of resistant bacteria (8–10). Although cutting-edge technologies–such as advanced oxidation processes like ozonation and photocatalysis, membrane filtration (e.g., reverse osmosis and nanofiltration), and activated carbon adsorption–are being explored for their ability to remove antibiotics, no single method has proven entirely effective (11–13). As a result, wastewater can play a significant role in the spread of AMR and has gotten increased attention lately (14, 15).

Wastewater serves as a rich and diverse habitat for a wide combination of environmental microorganisms, including bacteria, protozoa, fungi, and even viruses. These microorganisms often form complex communities, with biofilms being a particularly common feature that contributes to an ecosystem that allows all these microorganisms to coexist (16, 17). These biofilms feed on the nutrients and contaminants present in wastewater and can help degrade and remove several components, such as nitrogen and phosphorus compounds, complex organic compounds, and even heavy metals (18). For that reason, many WWTPs include biofilm-based biological treatments to remove contaminants, mainly due to their potential to provide practical, low-cost, and environmentally friendly solutions over chemical treatments (19, 20). However, although biological systems in WWTPs are initially intended to remove contaminants, an underexplored challenge presented in these biofilm-based systems is their capacity to protect potential pathogens and enhance the transfer of ARGs, making them ideal settings for the development and spread of AMR (9). In particular, the high cell density present in biofilms promotes close interactions between bacteria, facilitating gene exchange through conjugation at a faster rate and greater efficiency than planktonic cultures (21–23). Common biofilm-forming bacteria that can develop in wastewater include *Staphylococcus aureus, Klebsiella pneumoniae,* or *Pseudomonas aeruginosa*, which are considered some of the most relevant multidrug-resistant pathogens associated with mortality and morbidity around the globe (24). However, past research in this area has primarily focused on AMR in biofilms downstream of wastewater sites, particularly in freshwater biofilm communities (25–28), with comparatively little attention given to biofilms within wastewater systems themselves (29, 30).

### 1.2 AMR in vulnerable settings

The misuse and inappropriate prescribing of antibiotics in vulnerable settings such as refugee camps or migrant shelters have resulted in a global crisis of antibiotic resistance due to limited access to healthcare, lack of regulation, and poor sanitation and hygiene in these regions (31). At the same time, 75% of forcibly displaced persons are housed in low and middle-income countries (LMIC), which frequently face challenges related to AMR (32). In addition, many migrant shelters often lack adequate wastewater treatment facilities and infrastructure, and the surface drainage that most camps have typically results in water stagnation. These issues often lead to increased bacterial growth and create ideal conditions for biofilm development, potentially increasing the rates of AMR (33). These factors, when taken together, showcase the heightened risk that vulnerable and marginalized populations face across the globe. The severity of AMR challenges in displacement contexts has also been increasingly studied in recent years. For instance, studies have revealed an increased prevalence of multidrug-resistant organisms in refugee camps in the Middle East and Sub-Saharan Africa, often linked to inadequate water, sanitation, and hygiene infrastructures and limited healthcare access (34). Similarly, past research has shown that in crisis-affected communities, AMR hotspots often emerge at the intersection of overcrowding, poor sanitation, and limited practices to promote the responsible use of antibiotics. This highlights the urgent need to improve surveillance tools like biofilm sampling in these high-risk environments (35).

Health monitoring strategies can vary by location and resources, often more commonly implemented in high-income regions than in vulnerable communities of LMIC. For instance, a study on Lebanon, Jordan, and Uganda found that all relied on modeling rather than local data collection, highlighting the need for standardized practices to better understand refugee health (36). Similarly, the U.S.-Mexico border region faces disparities in wastewater infrastructure usually exist between twin cities like Ciudad Juárez-El Paso and Tijuana-San Diego, commonly leading to inconsistent treatment processes and frequent discharge of untreated or poorly treated wastewater, ultimately increasing environmental contamination of shared water resources such as the Rio Grande (37, 38). Many other areas along the border also lack comprehensive WWTPs, increasing public health risks. This affects vulnerable communities on both sides of the border, including migrant shelters and underserved rural areas, where exposure to pathogens and pollutants increases the spread of drug-resistant infections. Given the limited efficiency of antibiotics and rapid infection rates within these communities, biofilms formed in the surrounding wastewater are likely to harbor and protect high concentrations of pathogenic bacteria, increasing the rates of AMR development (39).

### 1.3 The need for wastewater monitoring: going beyond COVID-19

Due to the presence of high concentrations of microorganisms and contaminants, as well as resistance patterns in wastewater, monitoring it more closely becomes crucial for understanding and managing population health risks. In this context, wastewater surveillance, also known as wastewater-based epidemiology (WBE), is a valuable decision-support tool mainly intended to monitor municipal, clinical, and industrial sewage. With a primary focus on monitoring infectious diseases, WBE enables early detection of potential outbreaks in a specific region, even before individuals can show symptoms, allowing for early intervention and control (40). For instance, WBE emerged as a vital tool during the COVID-19 pandemic, offering non-invasive monitoring of community-level infection trends and early warning of outbreaks, often before clinical cases were reported (41). Its success has also highlighted the potential for broader applications in monitoring other public health threats, such as the growing AMR, as it can be implemented to detect and monitor the presence of ARGs over time and in different regions to further implement improved treatments or policies to mitigate AMR (40).

There are currently no standardized or specific methodologies for conducting WBE to monitor AMR. Common practices include single or a 24-h sample collection–grab or composite sampling, respectively–or taken from various locations within the same wastewater stream, providing a snapshot of AMR status at a specific point in time (42). A significant challenge present in these types of WBE for monitoring AMR is that the samples are generally affected by variations in wastewater flow rates. Storms or excessive rainfall can dilute samples or increase flow rates, reducing hydraulic retention times and temporarily washing out slow-growing microorganisms, obscuring trends in detecting antibiotic-resistant bacteria or ARGs. Additionally, and particularly in migrant shelters, the population tends to fluctuate rapidly, with individuals staying only temporarily. This constant population movement can lead to rapid changes in AMR patterns, making it challenging to capture consistent trends. Therefore, it is essential to implement a more comprehensive sampling methodology to accurately monitor AMR, ensuring that the dynamic nature of these settings is reflected in the results. Furthermore, WBE can also emerge not only for tracking AMR trends but also as a valuable tool to better understand the mechanisms driving the rise of AMR in wastewater.

## 2 A more robust WBE strategy: introducing biofilm sampling

Despite their critical role in AMR, current WBE methodologies largely overlook biofilm sampling, potentially underestimating the total concentration of ARGs in wastewater systems. This omission may represent a significant gap in the true extent of AMR spread within the environment. Since resistance mutations require time to establish and propagate, ARGs in traditional WBE samples do not necessarily imply acquired resistance in a host or the development of resistant bacterial strains (24). However, as biofilms are inherently providers of ideal environments for the persistence and retention of microorganisms, their presence ensures the required time for growth and establishment of large enough groups of microorganisms. Biofilm’s long-term protection features also help prevent sampling dilution or washout of slow-growing microorganisms during increased flow rates caused by heavy rainfall, increasing the retention times of microorganisms. This makes biofilms particularly useful in tracking different types of resistant organisms, especially in systems where environmental variability may otherwise obscure critical data, as it is less influenced by temporary changes in wastewater composition and flow. Biofilms can also provide more significant concentrations of DNA material than planktonic cells in standard WBE sampling techniques due to the number of cells present in biofilm clusters, helping to an early detection of ARB with low sample concentrations, which might not be possible through routine sampling of water or environmental surfaces alone. Consequently, the cell protection advantages that biofilms provide, along with their capability to retain diverse microbial communities and the large numbers of cell-to-cell interactions, allow for higher rates of exchange of genetic materials.

**Figure 1:**
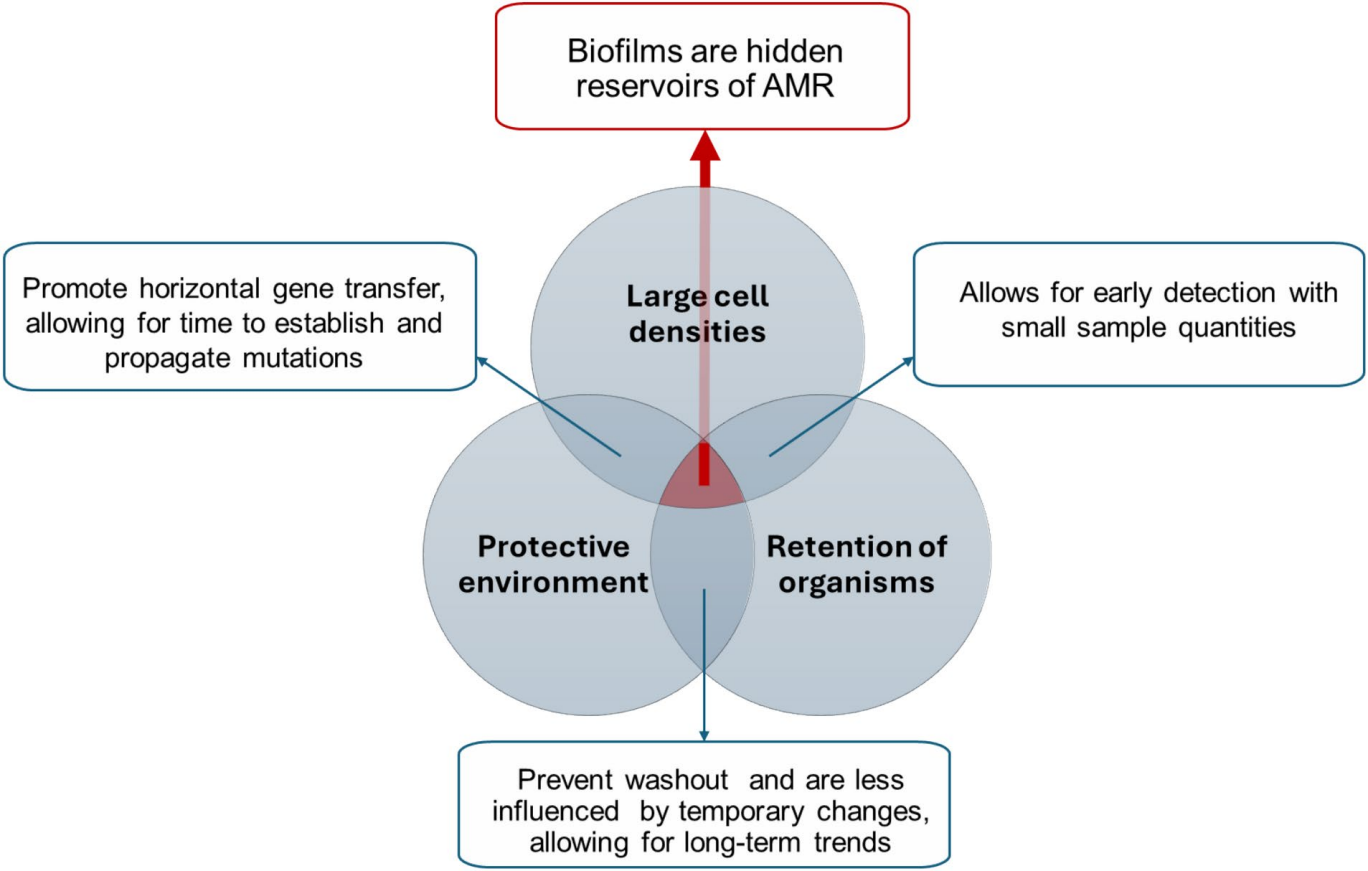
Biofilms as robust tools for AMR detection.

Figure 1 summarizes the key factors that make biofilms essential in the development of AMR; the combination of high cell densities, along with the protective environment provided by biofilms EPS matrix, allows for long-term retention of organisms, enhances horizontal gene transfer and prevents washout, enabling the long-term persistence of AMR. Therefore, biofilms may constitute hidden or understudied long-term reservoirs of resistance, potentially contributing to an underexplored public health threat. Introducing biofilm sampling into WBE may thereby allow for a more accurate estimate of the status of AMR and offer a more reliable and robust strategy compared to current WBE methodologies.

In contrast to conventional WBE approaches that focus primarily on planktonic bacteria in a given moment, biofilm-based surveillance may offer a more stable and integrative perspective of AMR. Because biofilms accumulate and retain microorganisms over time, they provide a continuous record of resistance dynamics, capturing core communities less affected by short-term fluctuations in wastewater composition (43). This enables the detection of low-prevalence ARGs that might be missed in standard grab samples and offers insights into long-term trends, bridging the gap between transient signals and the underlying, persistent reservoirs of resistance (44).

### 2.1 Biofilm sampling near migrant and refugee settlements

Biofilm WBE could be especially valuable in migrant shelters and refugee camps, where biofilm formation and increased AMR are likely to occur. In addition, these dynamic settings experienced in migrant shelters due to frequent movement and population variability complicate the tracking of AMR trends. Here, biofilms offer a relatively stable environment for microorganisms, enabling the consistent collection of AMR data and resistant bacteria despite changes in the population. In addition, given the lack of proper infrastructure or climate disasters such as extreme flooding, these communities might be likely affected at a higher scale than other settings. Sampling biofilms in these cases could help capture resistant bacteria and ARG that may otherwise be highly diluted or washed out, preventing the capture of accurate trends. In addition, inadequate waste management and wastewater treatment are common issues in vulnerable communities. The surrounding environment is commonly exposed to significant concentrations and types of contaminants such as heavy metals, microplastics, and pharmaceutical residues. Recent studies suggest that these contaminants may contribute to the increased AMR observed in wastewater (45–47). Although the precise mechanisms are not yet fully understood, biofilms may play a key role in AMR dynamics in these environments due to their active interactions with wastewater components. Therefore, a biofilm-based WBE approach could be critical for understanding how AMR interacts with contaminants and spreads in such vulnerable settings.

An accurate and effective implementation of biofilm-based WBE needs standardized sampling protocols, including careful selection of sampling sites (e.g., pipe surfaces, sediment traps, or biofilm carriers in treatment reactors) and consistent sampling frequency to account for seasonality and hydraulic variability. Techniques such as employing removable coupons or scraping biofilm from defined areas can yield replicable samples, while molecular tools like qPCR or metagenomics can facilitate ARG detection and quantification (48). The limited resource availability and the complexity of low-income settings, along with biofilm complexity and heterogeneity, may pose challenges, emphasizing the need for simple and cost-effective methods, as well as training for local personnel to ensure accurate and sustainable sampling efforts (49).

### 2.2 Ethical and social dimensions

All research and implementation of biofilm-based surveillance in vulnerable settings, particularly among people on the move, must adhere to strict ethical standards to avoid stigma and discrimination. Data collection and analysis should prioritize improving living conditions, access to healthcare, and infrastructure for affected populations. Therefore, prior engagement and agreement of community members are essential to understanding the root causes of AMR within migrant shelters and developing effective strategies to mitigate it (50). Biofilm-based WBE involves sampling near communities that often lack agency in surveillance decisions. Therefore, informed consent and transparent communication about the research’s purpose, methods, and intended benefits are crucial to building trust and avoiding stigmatization (51).

The data obtained can help guide actionable interventions, such as improvement of infrastructure and treatment protocols or better practices of antibiotics usage, ensuring that surveillance efforts translate into tangible health benefits for the affected populations.

## 3 Conclusion

AMR is a growing global concern that threatens public health and disproportionately affects vulnerable communities, including locations that host migrants and refugees. Within this context, biofilms are significant contributors to the persistence and spread of AMR, acting as key reservoirs of antibiotic-resistant genes in wastewater environments. Despite this, the role of biofilms in the spread of AMR has often been overlooked. Regular monitoring and control of ARG occurrence in biofilms should be prioritized, especially in vulnerable settings, current hotspots for AMR, and where biofilm development may accelerate dissemination rates.

Biofilm sampling can offer a unique advantage in tracking resistance, as it can capture the adaptive responses of microbial communities and ARGs despite rapid environmental changes, potentially providing a more accurate snapshot of resistant pathogens present in wastewater systems, compared to conventional surveillance strategies. Additionally, the high cell density within biofilms enables the effective detection of ARGs even at low concentrations, requiring smaller sample volumes than traditional methods. Furthermore, the significantly higher concentration of ARGs in biofilms compared to water samples suggests a more accurate and representative assessment of AMR. Therefore, biofilm-based monitoring could serve as a more robust and reliable approach for tracking AMR trends in wastewater. We encourage the scientific, public health and global health security community to explore biofilm-targeted approaches in wastewater surveillance, as they can help better understand and manage the adaptive processes that drive the spread of AMR in critical and vulnerable environments and mitigate global risk, while still protecting vulnerable communities.

## Data Availability

The original contributions presented in the study are included in the article/supplementary material, further inquiries can be directed to the corresponding author.

## References

[1] Roca I Akova M Baquero F Carlet J Cavaleri M Coenen S. The global threat of antimicrobial resistance: science for intervention. New Microbes New Infect. (2015) 6:22–9. doi: 10.1016/j.nmni.2015.02.007, PMID: 26029375 PMC4446399

[2] Tiwari A Kurittu P al-Mustapha AI Heljanko V Johansson V Thakali O. Wastewater surveillance of antibiotic-resistant bacterial pathogens: a systematic review. Front Microbiol. (2022) 13:977106. doi: 10.3389/fmicb.2022.977106, PMID: 36590429 PMC9798455

[3] Holvoet K Sampers I Callens B Dewulf J Uyttendaele M. Moderate prevalence of antimicrobial resistance in *Escherichia coli* isolates from lettuce, irrigation water, and soil. Appl Environ Microbiol. (2013) 79:6677–83. doi: 10.1128/AEM.01995-13, PMID: 23974140 PMC3811515

[4] Essigmann HT Aguilar DA Perkison WB Bay KG Deaton MR Brown SA. Epidemiology of antibiotic use and drivers of cross-border procurement in a Mexican American border community. Front Public Health. (2022) 10:832266. doi: 10.3389/fpubh.2022.832266, PMID: 35356027 PMC8960039

[5] Flemming HC Wingender J. The biofilm matrix. Nat Rev Microbiol. (2010) 8:623–33. doi: 10.1038/nrmicro2415, PMID: 20676145

[6] Madsen JS Burmølle M Hansen LH Sørensen SJ. The interconnection between biofilm formation and horizontal gene transfer. FEMS Immunol Med Microbiol. (2012) 65:183–95. doi: 10.1111/j.1574-695X.2012.00960.x, PMID: 22444301

[7] Wei Q Ma LZ. Biofilm matrix and its regulation in *Pseudomonas aeruginosa*. Int J Mol Sci. (2013) 14:20983–1005. doi: 10.3390/ijms141020983, PMID: 24145749 PMC3821654

[8] Read DS Gweon HS Bowes MJ Anjum MF Crook DW Chau KK. Dissemination and persistence of antimicrobial resistance (AMR) along the wastewater-river continuum. Water Res. (2024) 264:122204. doi: 10.1016/j.watres.2024.122204, PMID: 39116608 PMC7617467

[9] Sambaza SS Naicker N. Contribution of wastewater to antimicrobial resistance: a review article. J Glob Antimicrob Resist. (2023) 34:23–9. doi: 10.1016/j.jgar.2023.05.010, PMID: 37285914

[10] Zhang Y Marrs CF Simon C Xi C. Wastewater treatment contributes to selective increase of antibiotic resistance among *Acinetobacter* spp. Sci Total Environ. (2009) 407:3702–6. doi: 10.1016/j.scitotenv.2009.02.013, PMID: 19321192

[11] Dong H Yuan X Wang W Qiang Z. Occurrence and removal of antibiotics in ecological and conventional wastewater treatment processes: a field study. J Environ Manag. (2016) 178:11–9. doi: 10.1016/j.jenvman.2016.04.037, PMID: 27127893

[12] Mouele ESM Tijani JO Badmus KO Pereao O Babajide O Zhang C. Removal of pharmaceutical residues from water and wastewater using dielectric barrier discharge methods—a review. Int J Environ Res Public Health. (2021) 18:1683. doi: 10.3390/ijerph18041683, PMID: 33578670 PMC7916394

[13] Phoon BL Ong CC Mohamed Saheed MS Show PL Chang JS Ling TC. Conventional and emerging technologies for removal of antibiotics from wastewater. J Hazard Mater. (2020) 400:122961. doi: 10.1016/j.jhazmat.2020.122961, PMID: 32947727

[14] Amador PP Fernandes RM Prudêncio MC Barreto MP Duarte IM. Antibiotic resistance in wastewater: occurrence and fate of Enterobacteriaceae producers of class A and class C β-lactamases. J Environ Sci Health A. (2015) 50:26–39. doi: 10.1080/10934529.2015.964602, PMID: 25438129

[15] Karkman A Do TT Walsh F Virta MPJ. Antibiotic-resistance genes in waste water. Trends Microbiol. (2018) 26:220–8. doi: 10.1016/j.tim.2017.09.005, PMID: 29033338

[16] Flemming HC Wingender J Szewzyk U Steinberg P Rice SA Kjelleberg S. Biofilms: an emergent form of bacterial life. Nat Rev Microbiol. (2016) 14:563–75. doi: 10.1038/nrmicro.2016.94, PMID: 27510863

[17] Røder HL Olsen NMC Whiteley M Burmølle M. Unravelling interspecies interactions across heterogeneities in complex biofilm communities. Environ Microbiol. (2020) 22:5–16. doi: 10.1111/1462-2920.14834, PMID: 31637837

[18] Verma S Kuila A Jacob S. Role of biofilms in waste water treatment. Appl Biochem Biotechnol. (2023) 195:5618–42. doi: 10.1007/s12010-022-04163-5, PMID: 36094648

[19] Dhanasekaran D Thajuddin N. Microbial biofilms: importance and applications. Croatia, IntechOpen: BoD – Books on Demand (2016).

[20] Saini S Tewari S Dwivedi J Sharma V. Biofilm-mediated wastewater treatment: a comprehensive review. Mater Adv. (2023) 4:1415–43. doi: 10.1039/D2MA00945E

[21] Angles ML Marshall KC Goodman AE. Plasmid transfer between marine Bacteria in the aqueous phase and biofilms in reactor microcosms. Appl Environ Microbiol. (1993) 59:843–50. doi: 10.1128/aem.59.3.843-850.1993, PMID: 16348893 PMC202198

[22] Guo X Yang Y Lu DP Niu ZS Feng JN Chen YR. Biofilms as a sink for antibiotic resistance genes (ARGs) in the Yangtze estuary. Water Res. (2018) 129:277–86. doi: 10.1016/j.watres.2017.11.029, PMID: 29156392

[23] Michaelis C Grohmann E. Horizontal gene transfer of antibiotic resistance genes in biofilms. Antibiotics. (2023) 12:328. doi: 10.3390/antibiotics12020328, PMID: 36830238 PMC9952180

[24] Marano RBM Gupta CL Cozer T Jurkevitch E Cytryn E. Hidden Resistome: enrichment reveals the presence of clinically relevant antibiotic resistance determinants in treated wastewater-irrigated soils. Environ Sci Technol. (2021) 55:6814–27. doi: 10.1021/acs.est.1c00612, PMID: 33904706

[25] Kaeseberg T Schubert S Oertel R Zhang J Berendonk TU Krebs P. Hot spots of antibiotic tolerant and resistant bacterial subpopulations in natural freshwater biofilm communities due to inevitable urban drainage system overflows. Environ Pollut. (2018) 242:164–70. doi: 10.1016/j.envpol.2018.06.081, PMID: 29980034

[26] Matviichuk O Mondamert L Geffroy C Gaschet M Dagot C Labanowski J. River biofilms microbiome and Resistome responses to wastewater treatment plant effluents containing antibiotics. Front Microbiol. (2022) 13:795206. doi: 10.3389/fmicb.2022.795206, PMID: 35222329 PMC8863943

[27] Proia L von Schiller D Sànchez-Melsió A Sabater S Borrego CM Rodríguez-Mozaz S. Occurrence and persistence of antibiotic resistance genes in river biofilms after wastewater inputs in small rivers. Environ Pollut. (2016) 210:121–8. doi: 10.1016/j.envpol.2015.11.035, PMID: 26708766

[28] Zhu Y-G Zhao Y Li B Huang CL Zhang SY Yu S. Continental-scale pollution of estuaries with antibiotic resistance genes. Nat Microbiol. (2017) 2:16270–7. doi: 10.1038/nmicrobiol.2016.270, PMID: 28134918

[29] Lépesová K Kraková L Pangallo D Medveďová A Olejníková P Mackuľak T. Prevalence of antibiotic-resistant coliform bacteria, Enterococcus spp. and Staphylococcus spp. in wastewater sewerage biofilm. J Glob Antimicrob Resist. (2018) 14:145–51. doi: 10.1016/j.jgar.2018.03.008, PMID: 29604432

[30] Subirats J Triadó-Margarit X Mandaric L Acuña V Balcázar JL Sabater S. Wastewater pollution differently affects the antibiotic resistance gene pool and biofilm bacterial communities across streambed compartments. Mol Ecol. (2017) 26:5567–81. doi: 10.1111/mec.14288, PMID: 28792642

[31] Kamiab Hesari D Aljadeeah S Brhlikova P Hyzam D Komakech H Patiño Rueda JS. Access to and utilisation of antimicrobials among forcibly displaced persons in Uganda, Yemen and Colombia: a pilot cross-sectional survey. BMJ Open. (2024) 14:e084734. doi: 10.1136/bmjopen-2024-084734, PMID: 39013652 PMC11253744

[32] Pokharel S Raut S Adhikari B. Tackling antimicrobial resistance in low-income and middle-income countries. BMJ Glob Health. (2019) 4:e002104. doi: 10.1136/bmjgh-2019-002104, PMID: 31799007 PMC6861125

[33] Ajibade O Tota-Maharaj K Clarke B. Challenges of poor surface water drainage and wastewater management in refugee camps. Environ Earth Sci Res J. (2016) 3:53–60. doi: 10.18280/eesrj.030402

[34] Van Der Bij AK Pitout JDD. The role of international travel in the worldwide spread of multiresistant Enterobacteriaceae. J Antimicrob Chemother. (2012) 67:2090–100. doi: 10.1093/jac/dks214, PMID: 22678728

[35] Topluoglu S Taylan-Ozkan A Alp E. Impact of wars and natural disasters on emerging and re-emerging infectious diseases. Front Public Health. (2023) 11:1215929. doi: 10.3389/fpubh.2023.1215929, PMID: 37727613 PMC10505936

[36] Bou-Karroum L Daher N Jabbour M Akhu-Zaheya L Khater W Alloubani A. Assessing the integration of refugee health data into national health information systems in Jordan, Lebanon, and Uganda. Confl Heal. (2024) 18:49. doi: 10.1186/s13031-024-00608-2, PMID: 39103863 PMC11299268

[37] Carrillo G Uribe F Lucio R Lopez AR Korc M. The United States-Mexico border environmental public health: the challenges of working with two systems. Rev Panam Salud Publica. (2017) 41:e98. doi: 10.26633/RPSP.2017.98, PMID: 28902281 PMC6660844

[38] Moya E. M. Solis G. Ramos R. L. Lusk M. W. Quist C. S., “US–Mexico border: challenges and opportunities in rural and border health,” In Rural nursing: Concepts, theory, and practice, Fourth Edition, Springer Publishing Company, (2013), pp. 303–333. Available at: https://books.google.com/books?id=W14JcBPzguAC.

[39] Fuentes MD Gutierrez S Sahagun D Gomez J Mendoza J Ellis CC. Assessment of antibiotic levels, multi-drug resistant Bacteria and genetic biomarkers in the waters of the Rio Grande River between the United States-Mexico border. J Health Pollut. (2019) 9:190912. doi: 10.5696/2156-9614-9.23.190912, PMID: 31497375 PMC6711330

[40] Chau KK Barker L Budgell EP Vihta KD Sims N Kasprzyk-Hordern B. Systematic review of wastewater surveillance of antimicrobial resistance in human populations. Environ Int. (2022) 162:107171. doi: 10.1016/j.envint.2022.107171, PMID: 35290866 PMC8960996

[41] Harrington A Vo V Papp K Tillett RL Chang CL Baker H. Urban monitoring of antimicrobial resistance during a COVID-19 surge through wastewater surveillance. Sci Total Environ. (2022) 853:158577. doi: 10.1016/j.scitotenv.2022.158577, PMID: 36087661 PMC9450474

[42] Huijbers PMC Bobis Camacho J Hutinel M Larsson DGJ Flach C-F. Sampling considerations for wastewater surveillance of antibiotic resistance in fecal Bacteria. Int J Environ Res Public Health. (2023) 20:4555. doi: 10.3390/ijerph20054555, PMID: 36901565 PMC10002399

[43] Newton RJ McLellan SL Dila DK Vineis JH Morrison HG Eren AM. Sewage reflects the microbiomes of human populations. MBio. (2015) 6:e02574. doi: 10.1128/mbio.02574-14 25714718 PMC4358014

[44] Sims N Kasprzyk-Hordern B. Future perspectives of wastewater-based epidemiology: monitoring infectious disease spread and resistance to the community level. Environ Int. (2020) 139:105689. doi: 10.1016/j.envint.2020.105689, PMID: 32283358 PMC7128895

[45] Sathicq MB Sabatino R Corno G Di Cesare A. Are microplastic particles a hotspot for the spread and the persistence of antibiotic resistance in aquatic systems? Environ Pollut. (2021) 279:116896. doi: 10.1016/j.envpol.2021.116896, PMID: 33744628

[46] Sutradhar I Kalyan P Chukwu K Abia ALK Mbanga J Essack S. Metal ions and their effects on antimicrobial resistance development in wastewater. bioRxiv. (2023). doi: 10.1101/2023.06.16.545339

[47] Wang H Xu K Wang J Feng C Chen Y Shi J. Microplastic biofilm: an important microniche that may accelerate the spread of antibiotic resistance genes via natural transformation. J Hazard Mater. (2023) 459:132085. doi: 10.1016/j.jhazmat.2023.132085, PMID: 37494793

[48] Fiorentino A di Cesare A Eckert EM Rizzo L Fontaneto D Yang Y. Impact of industrial wastewater on the dynamics of antibiotic resistance genes in a full-scale urban wastewater treatment plant. Sci Total Environ. (2019) 646:1204–10. doi: 10.1016/j.scitotenv.2018.07.370, PMID: 30235606

[49] Flemming H-C van Hullebusch ED Little BJ Neu TR Nielsen PH Seviour T. Microbial extracellular polymeric substances in the environment, technology and medicine. Nat Rev Microbiol. (2024) 23:87–105. doi: 10.1038/s41579-024-01098-y, PMID: 39333414

[50] Bowes DA Zaman MH. Considerations for conducting wastewater-based public health assessments in migrant populations. J Environ Expo Assess. (2023) 2:20. doi: 10.20517/jeea.2023.24

[51] Clark J Muñoz S Auerbach J. When top-down infrastructures fail: spaces and practices of care and community under COVID-19. Soc Cult Geogr. (2023) 24:542–62. doi: 10.1080/14649365.2022.2115119, PMID: 39989647

